# An automatic collector for density gradients

**DOI:** 10.1155/S1463924680000259

**Published:** 1980

**Authors:** Rex Mottershead

**Affiliations:** Medical Research Council Cell Mutation Unit University of Sussex Sussex Brighton BN1 9QG UK


### An automatic collector for density gradients

**Rex Mottershead,**
*Medical Research Council Cell Mutation Unit, University of Sussex, Brighton, Sussex BN1 9QG, UK.*

### Introduction
The paper strip method of Carrier et al for collecting density gradients [1] eliminated some of the tedium associated with the earlier filter disc technique of Bollum [2]. The automatic collector described here was developed to further improve the technique by removing the need for the operator to count drops or time or to move the strips by hand at short intervals. This has advantages in that it speeds the process considerably without loss of accuracy and further eliminates tedium for the operator.

### Description of collector
The principle component of the collector is a 'Minipump' type IV peristaltic pump made by Schuco Scientific Ltd., London, England. The pump is used to remove the contents of a density gradient and to dispense them onto a paper tape which is continuously advanced by a transport mechanism connected directly to the rotor of the pump (Figure 1).

The pump is attached to a base plate which also carries a holder for a reel of Whatman grade 17.1 cm cellulose paper tape. A short channel, 1cm wide, is mounted on the side of the plastic cover of the pump. The channel and the cover are drilled in line with the centre of the pump rotor and a short nylon rod, covered by silicone rubber tubing, is passed through the channel and engaged in the hole in the rotor where it is held by friction. A flat phosphor-bronze spring holds a small idler wheel against the underside of the rod, these two parts together forming the tape transport mechanism. The input of the peristaltic pump is connected by a short length of flexible tubing to a section of 1.3 mm o.d. cannula. The output is attached to a second short length of tubing, the end of which is held by a bracket over the end of the channel. Figure 2 shows the overall layout of the device.

### Operation of the collector
Operation of the collector is very simple; the section of cannula is lowered to the bottom of a prepared gradient, the paper tape is engaged in the transport mechanism and the machine is switched on. When the contents of the gradient have been transferred to the paper tape, the motor is switched off, the used tape is removed and the collector is then ready

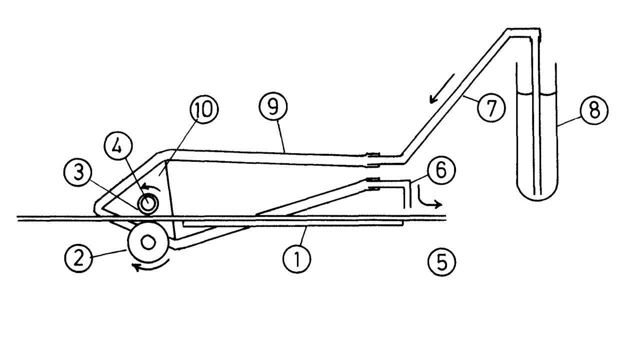

*Figure 1. Schematic diagram of the mode of operation of the collector. (see Figure 2. for key).*

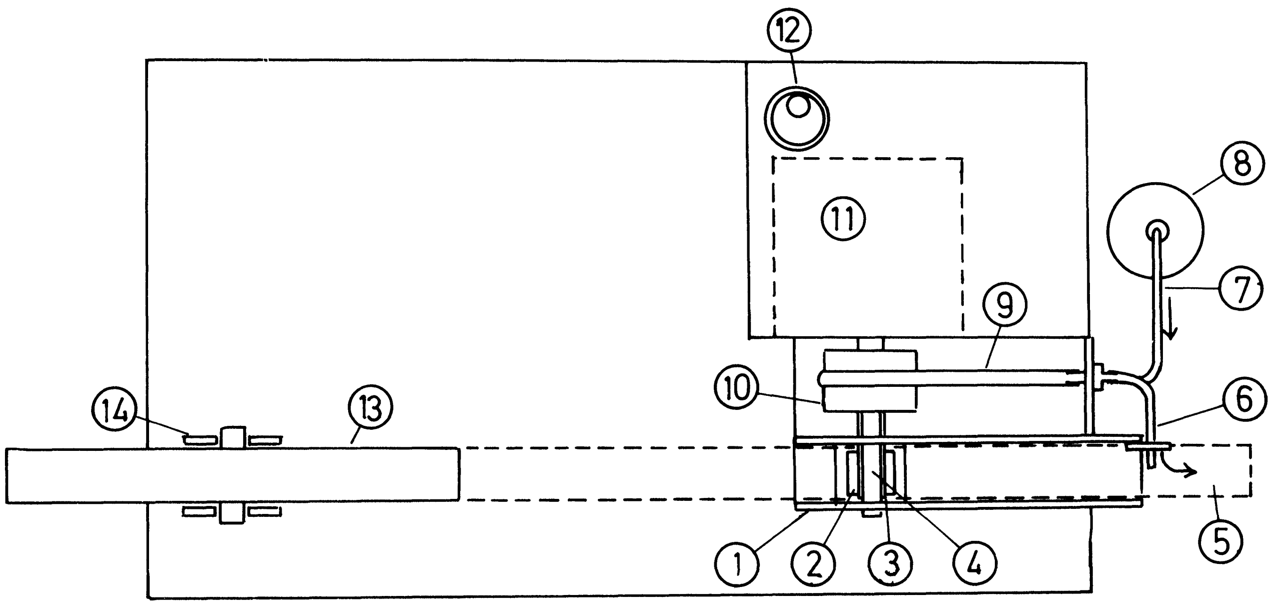

*Figure 2. Diagram showing general layout of machine – top view. (1) Guide channel. (2) Idler wheel (3) Silicone tubing. (4) Drive shaft. (5) Paper tape. (6) Outlet tube. (7) Inlet tube. (8) Density gradient. (9) Pump tubing. (10) Pump rotor. (11) Pump motor. (12) On-off switch. (13) Reel of paper tape. (14) Tape reel holder.*

for another gradient. After fixing and drying, the paper strip is marked off into equal lengths using a six-bladed roller mounted in a handle which was designed for the purpose.

Using silicone tubing in the pump of 2 mm inside diameter and a 3 mm diameter nylon rod covered by 5 mm outside diameter tubing forming the drive-shaft in the tape transport mechanism, a 5 ml gradient is distributed onto approximately 50 cm of paper tape in about 30 seconds. Experiments carried out by applying a layer of dye on top of a gradient during the collection process showed that no visible signs of distortion occurred using the automatic device.

### Comparability study

The machine has been used in the author's laboratory to collect gradients containing DNA from bacteria and from mammalian cells. For the comparability study, human fibroblasts were labelled for 24 hours with 1 $\mu$Ci/ml $^3$H thymidine and $\gamma$– irradiated. Duplicate 0.1 ml samples were lysed in 0.2 ml 2% sodium dodecyl sulphate/0.02M EDTA, which had previously been layered onto 4.7 ml alkaline sucrose gradients (5-20% sucrose, 0.1M NaCl, 0.1M NaOH). The gradients were centrifuged for 1 hour at 38,000 rpm in the SW50.1 rotor of a Beckman L2-65B ultracentrifuge. One of each pair was collected manually, the other by the machine. The resulting strips were washed twice each in 5% trichloracid and ethanol prior to estimation in an Intertechnique SL40 scintillation counter.

### Results

The results of the comparability study are shown in Figure 3. The profiles of a typical pair of gradients fractionated manually or automatically were very similar, and well within the general limits of reproducability expected from duplicate gradients collected the same way. Subsequent routine use of the machine in this laboratory for over a year has not revealed any fundamental problems, although minor faults have occasionally developed. These usually occur as a result of a blockage of the fine tubing used in the construction of the machine.

### Discussion

The manual methods of collecting density gradients onto strips of paper use either time or number of drops to control the volume of liquid placed on each pre-marked unit of paper. These methods are subject to errors caused by operator fatigue or distraction and are tedious. Many of these problems have been overcome by the use in some laboratories of multi-channel pumps to collect several gradients simultaneously with the strips moved by hand at fixed time intervals. This is rapid but still requires intense operator concentration for reliable performance. The collecting device described in this paper is rapid, does not require counting of drops or time, and is as accurate as manual methods. It therefore holds advantages over systems developed elsewhere.

ACKNOWLEDGEMENTS

I thank Ms Stevie Stevens for performing the comparability studies.

REFERENCES

[1] Carrier, W.L. and Setlow, R.B. *Analytical Biochemistry,* 1971, 43, 427.

[2] Bollum, F.J. in 'Procedures in Nucleic Acid Research' Eds. Cantoni G.L. and Davies D.R. 1966, Harper and Row, New York, pp.296.

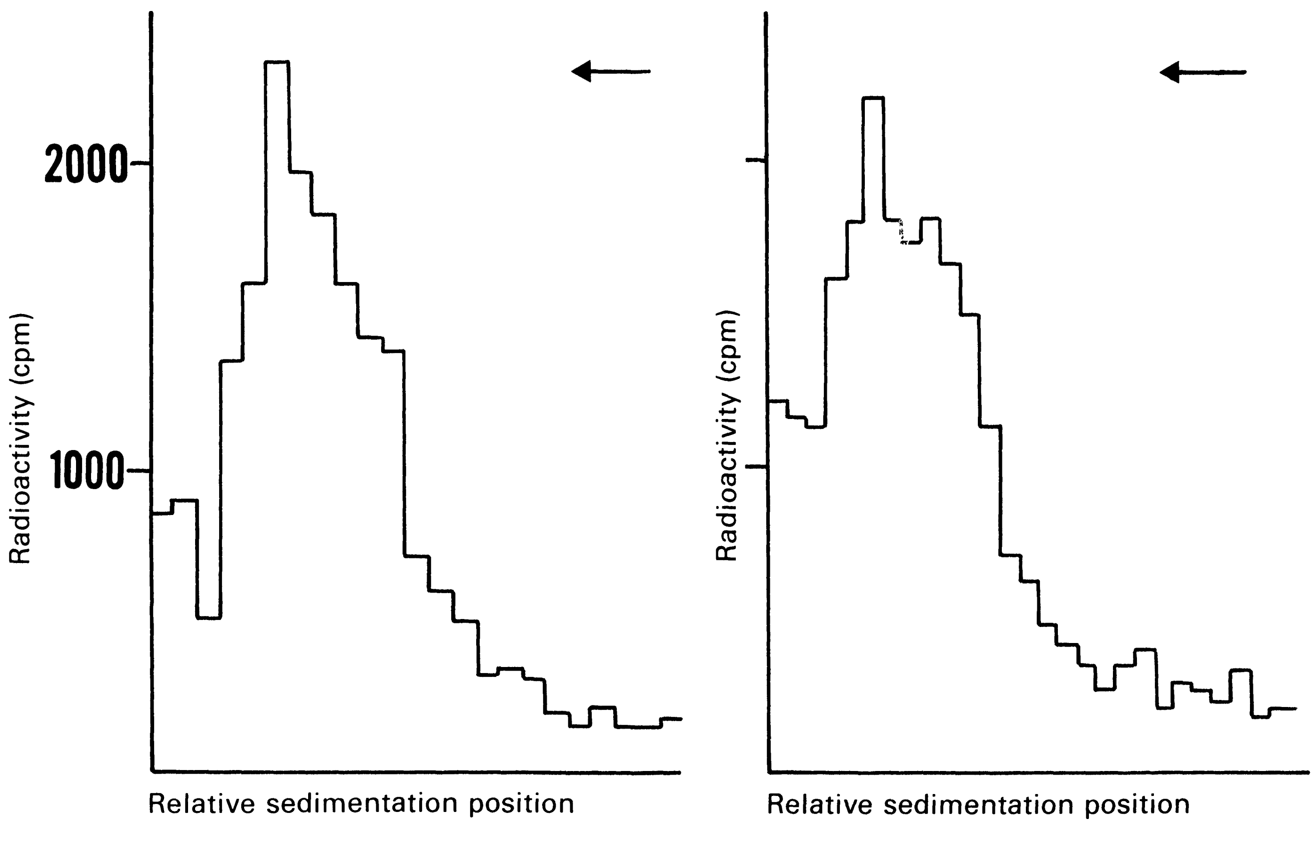

*Figure 3. Comparison of profiles resulting from manual collection (left) and automatic collection (right) of duplicate gradients. The ordinates indicate radioactivity in counts per minute in each fraction and abscissae indicate the relative sedimentation position of each fraction, the top of the gradient being to the right in each case. These results are typical of those obtained throughout the instrument's performance.*

